# Effects of Traffic-Related Diesel Exhaust Exposure and Moderate Exercise on Obesity, Metabolic Dysfunction, and Inflammatory Responses in Rats

**DOI:** 10.3390/metabo16090682

**Published:** 2026-09-16

**Authors:** Nesrullah Ayşin, Süheyla Altuğ Özsoy, Zübeyir Huyut

**Affiliations:** 1 Department of Medical Services and Techniques, Vocational School of Health Services, Hakkari University, Hakkari 30000, Türkiye; 2 Department of Public Health Nursing, Faculty of Nursing, Ege University, Izmir 35100, Türkiye; 3 Department of Biochemistry, Faculty of Medicine, Van Yuzuncu Yil University, Van 65080, Türkiye

**Keywords:** air pollution, diesel exhaust, PM2.5, obesity, exercise, inflammation, adipokines, metabolic disorders

## Abstract

Objective: Traffic-related air pollution is recognised as a significant environmental risk factor associated with obesity and metabolic dysfunction. Although the positive effects of regular exercise on metabolic health are well established, its protective role against traffic-related air-pollution-induced metabolic dysfunction has not been fully elucidated. This study was conducted to investigate the effects of diesel exhaust exposure and moderate exercise on the development of obesity, metabolic changes, adipokine profile, and inflammatory response in rats. Materials and Methods: In this randomised controlled experimental study, 48 female Wistar Albino rats were randomised into six experimental groups. The animals were exposed to diesel exhaust simulating traffic-related air pollution (average 300 μg PM2.5/m^3^) for 2 or 4 h daily over an eight-week period. Rats in the exercise groups underwent moderate-intensity treadmill exercise for 30 min at a speed of 15 m/min, five days a week, over the same period. At the end of the study, obesity indicators, serum lipid profile, glucose metabolism parameters, adipokines and inflammation markers were assessed. Results: Diesel exhaust exposure caused a significant increase in final body weight, BMI, Lee index and VAI values (*p* < 0.001). Furthermore, levels of LDL cholesterol, total cholesterol, triglycerides, glucose, adiponectin, leptin, CRP, IL-37, IL-1β, IL-6 and TNF-α increased significantly, whilst HDL cholesterol and insulin levels decreased significantly (*p* < 0.001). The most pronounced metabolic and inflammatory changes were observed in the 4 h exhaust gas exposure group. Elevations in pro-inflammatory cytokines, accompanied by increased visceral adiposity, supported the development of systemic metabolic inflammation. Exercise significantly reduced weight gain, visceral adiposity, dyslipidaemia, hyperglycaemia, adipokine imbalance and the inflammatory response (*p* < 0.001). However, exercise could not fully reverse all the changes associated with long-term diesel exhaust exposure. Conclusions: Traffic-related diesel exhaust exposure led to metabolic disorders in rats, characterised by visceral adiposity, dyslipidaemia, impaired glucose homeostasis, adipokine imbalance and chronic low-grade inflammation. Regular moderate-intensity exercise significantly reduced these adverse effects but could not eliminate them entirely. The findings suggest that traffic-related air pollution may contribute to the development of obesity via inflammation-mediated metabolic mechanisms, and that regular physical activity may serve as an important protective strategy in limiting these effects.

## 1. Introduction

Air pollution is currently recognised as one of the most significant environmental determinants of the global disease burden and is associated with millions of premature deaths each year [1,2]. Traffic-related air pollution (TRAP), particularly due to emissions from motor vehicles, has become a major public health issue in densely urbanised areas [3]. Traffic-related air pollution contains numerous toxic components, including fine particulate matter (PM2.5), nitrogen oxides, carbon monoxide, volatile organic compounds and polycyclic aromatic hydrocarbons [4,5]. In recent years, it has been demonstrated that these pollutants have significant effects not only on the respiratory and cardiovascular systems, but also on metabolic health [6].

Obesity is a chronic, multifactorial disease resulting from a disruption in energy homeostasis; it is regarded as a significant public health issue due to its rapidly increasing prevalence worldwide [7]. It has been suggested that the development of obesity cannot be explained solely by a high-calorie diet and physical inactivity, and that environmental exposures may also play a significant role in this process [8]. In particular, it has been reported that exposure to PM2.5 may have adverse effects on adipogenesis, lipid metabolism and glucose homeostasis [1].

Epidemiological and experimental studies indicate that long-term exposure to air pollution is associated with an increase in body mass index, abdominal adiposity, insulin resistance and metabolic syndrome [9]. It has been reported that PM2.5 exposure increases adipocyte hypertrophy, triggers inflammation in adipose tissue and disrupts energy metabolism [7]. Furthermore, it has been shown that fine particulate matter enters the circulation, causing oxidative stress, mitochondrial dysfunction and systemic inflammation; by affecting the secretion of adipokines such as leptin and adiponectin, it leads to disruptions in energy balance and metabolic processes [10,11,12].

Regular physical exercise, however, is recognised as one of the most effective lifestyle interventions for the prevention of obesity and metabolic diseases [13]. It is known that exercise increases glucose utilisation, supports lipid oxidation and suppresses inflammatory processes [14,15,16]. However, the metabolic effects of physical activity performed under conditions of traffic-related air pollution have not yet been fully elucidated [17,18]. In particular, there are only a limited number of experimental studies that have evaluated diesel exhaust exposure and exercise together [19,20]. Furthermore, important gaps remain regarding whether different durations of diesel exhaust exposure produce distinct obesity-related metabolic and inflammatory alterations and whether regular moderate-intensity exercise can attenuate these effects. The novelty of the present study lies in simultaneously comparing two different daily durations of diesel exhaust exposure (2 and 4 h/day) and evaluating the potential mitigating effects of exercise across multiple interconnected outcomes, including visceral adiposity, lipid and glucose metabolism, adipokine profiles, and systemic inflammatory markers.

The aim of this study was to investigate the effects of traffic-related diesel exhaust exposure and regular moderate-intensity exercise on the development of obesity in rats. In this context, changes in body weight, body mass index, Lee index, visceral adipose index, lipid profile, glucose metabolism, adipokines and inflammation markers were evaluated to examine the metabolic effects of traffic-related air pollution and the potential protective role of exercise.

## 2. Materials and Methods

In this randomised controlled experimental study, a total of 48 healthy, pathogen-free female Wistar Albino rats, aged 10–12 weeks and weighing 180–195 g, were obtained from the Van Yüzüncü Yıl University Experimental Animals Research and Application Center (Van, Türkiye). A single-sex experimental model was used to reduce potential variability associated with sex-related differences in metabolic and inflammatory responses, and female rats were selected for the present experimental protocol. Upon arrival, the animals were allowed to acclimatise to the laboratory environment before the initiation of the experimental procedures.

Throughout the study, rats were housed in standard polypropylene cages under controlled environmental conditions, including a temperature of 24 ± 3 °C, relative humidity of 50–60%, and a 12 h light/12 h dark cycle. Animals had ad libitum access to standard laboratory pellet chow and drinking water. Health status and general behaviour were monitored daily throughout the experimental period. The number of animals was determined before the experiment with reference to sample sizes used in previous comparable rodent studies of particulate matter and diesel exhaust exposure [21,22]. Eight animals were allocated to each of the six experimental groups (total *n* = 48), taking into consideration both the need for adequate group-level comparisons and the Reduction principle of the 3Rs.

Rats assigned to the exercise groups underwent a 7-day treadmill familiarisation period before the intervention phase. During this adaptation period, treadmill speed and duration were gradually increased to minimise stress and ensure compliance with the exercise protocol.

The study was conducted and reported in accordance with the ARRIVE guidelines. Ethical approval was obtained from the Van Yüzüncü Yıl University Local Animal Experimentation Ethics Committee (Approval No: 2022/01-14; Approval Date: 27 January 2022).

### 2.1. Experimental Procedure

Rats were randomly allocated into six groups (*n* = 8 per group) using a computer-generated random number table. The experimental conditions of each group are summarised in Table 1. Rats assigned to exercise underwent moderate-intensity treadmill exercise at 15 m/min for 30 min/day, 5 days/week, for 8 weeks. In the combined diesel exhaust and exercise groups, exercise was performed after completion of the daily exposure.

All exposure and exercise interventions were conducted 5 days/week for 8 weeks. In the combined groups, exercise was performed following the daily diesel exhaust exposure.

In the combined exposure and exercise groups, treadmill exercise was performed after completion of the daily diesel exhaust exposure. This sequence was selected to evaluate the potential mitigating effect of exercise following a defined pollution exposure while avoiding exercise-induced increases in minute ventilation during exposure, which could have altered the effective inhaled dose. Because exercise was initiated only after removal from the exposure chamber, increased ventilation during exercise did not directly increase pollutant inhalation.

### 2.2. Traffic-Related Air Pollution Exposure System

To simulate traffic-related diesel exhaust exposure, researchers set up a specialised exposure apparatus [23]. The system was designed based on the principle of transferring emissions from a diesel engine exhaust into the exposure chamber via a closed transfer line. Exhaust was generated using a four-stroke, 6.0 kW, 406 cc diesel generator operated with standard diesel fuel. The generator produced a total exhaust flow of approximately 580 L/min, of which approximately 19 L/min was directed through an insulated transfer line into the exposure system. Engine load was varied from idle to 2.5 kW in 0.5 kW increments using a SimplX Swift-E FT load bank. The exhaust underwent a two-stage dilution process. During the primary dilution stage, raw exhaust was mixed with filtered compressed air at a fixed dilution ratio of 9:1. Secondary dilution was achieved using HEPA-filtered air, with an average dilution ratio of approximately 25:1, and was adjusted to maintain the target PM2.5 concentration of approximately 300 μg/m^3^. A custom air mixer was positioned downstream of the secondary dilution system to promote homogeneous mixing before the diluted exhaust entered the exposure chamber. The walls of the exposure chamber were made of polycarbonate material to minimise particle adhesion, and the system was cleaned after each application. Airflow and chamber pressure were controlled using intake and exhaust fans to ensure a stable flow of diluted exhaust into the chamber. PM2.5 levels were continuously monitored using an aerosol particle monitor (DustTrak II Aerosol Monitor 8530, TSI Inc., Shoreview, MN, USA), and the exposure concentration was standardised to an average of 300 μg PM2.5/m^3^. In the control group, similar environmental conditions were maintained using HEPA-filtered clean air within the same exposure system. The exposure duration and concentration were selected with reference to previous controlled inhalation studies in rodents, in which diesel exhaust exposure for approximately 4 h/day has been widely used to induce measurable biological responses under experimental conditions [24,25,26,27,28]. The 4 h exposure was therefore used as the higher daily exposure condition, while a 2 h exposure group was included to allow comparison of responses according to daily exposure duration. Gas-phase co-pollutants, including nitrogen oxides (NOx) and carbon monoxide (CO), were not separately quantified during the exposure protocol; therefore, exposure characterisation was based on continuously monitored PM2.5 mass concentration.

For contextual comparison, exposure to 300 μg PM_2.5_/m^3^ would correspond to an estimated inhaled PM_2.5_ mass of approximately 500 μg over 2 h and 1000 μg over 4 h in a 70 kg adult, assuming an inhalation volume of 20 m^3^/day.

### 2.3. Sample Collection and Biochemical Analyses

Throughout the experiment, the rats’ body weights were measured weekly using a precision digital scale and recorded. At the end of the experimental period, nose-to-anus length was measured as body length using a non-stretchable measuring tape. Body mass index (BMI) was calculated as body weight (g)/[nose-to-anus length (cm)]^2^, and the Lee index was calculated as [cube root of body weight (g)/nose-to-anus length (cm)] × 10. Following euthanasia, abdominal adipose tissues, including retroperitoneal, epididymal, and omental fat depots, were dissected and weighed. The visceral adipose index (VAI) was calculated as [total abdominal adipose tissue weight (g)/final body weight (g)] × 100. These calculations were performed according to the procedures described in the original experimental protocol.

Following completion of the experimental procedures, all rats were deeply anaesthetised by intraperitoneal administration of ketamine (90 mg/kg). The depth of anaesthesia was confirmed by the absence of pedal withdrawal and corneal reflexes. Blood samples were subsequently collected by intracardiac puncture, and euthanasia was completed by exsanguination under deep anaesthesia. Death was confirmed by the absence of heartbeat, respiration, and reflex responses. All procedures were performed by trained personnel in accordance with institutional animal welfare regulations.

Blood samples were collected into dry vacuum gel tubes and centrifuged at 3500 rpm at 4 °C for 15 min to separate the serum. The separated serum samples were stored at −80 °C until biochemical analysis. Serum high-density lipoprotein (HDL), low-density lipoprotein (LDL), total cholesterol, triglyceride, glucose, and insulin levels were analysed using an automated biochemical analyser. Serum leptin and adiponectin concentrations were measured using commercially available rat-specific enzyme-linked immunosorbent assay (ELISA) kits (BT LAB; leptin, Cat. No. E0561Ra; adiponectin, Cat. No. E0758Ra) according to the manufacturer’s protocols.

Serum C-reactive protein (CRP) concentrations were determined using an Abbott Architect c16000 automated analyser (Abbott Laboratories, Abbott Park, IL, USA) with the Abbott Reagent Architect CRP kit (Kit No. 729084), according to the manufacturer’s instructions. Serum interleukin-37 (IL-37), interleukin-1β (IL-1β), interleukin-6 (IL-6), and tumour necrosis factor-alpha (TNF-α) concentrations were measured using commercially available rat-specific ELISA kits (BT LAB; IL-37, Cat. No. E2576Ra; IL-1β, Cat. No. E0119Ra; IL-6, Cat. No. E0135Ra; TNF-α, Cat. No. E0764Ra) according to the manufacturer’s protocols. The cytokine assays were based on the sandwich ELISA principle. Briefly, serum samples and standards were processed in antibody-coated microplates, followed by incubation with biotinylated detection antibodies and streptavidin–horseradish peroxidase (HRP). Following the prescribed incubation and washing steps, substrate solutions were added, and the reactions were terminated using stop solution. Optical density was measured at 450 nm within 15 min after addition of the stop solution, and cytokine concentrations were calculated from the corresponding standard curves.

### 2.4. Ethical Considerations

This study was approved by the Van Yüzüncü Yıl University Animal Experiments Local Ethics Committee (Approval No: 2022/01-14; Date: 27 January 2022). All experimental procedures were conducted in accordance with national regulations governing the care and use of laboratory animals and complied with the ARRIVE guidelines. Throughout the study, all efforts were made to minimise animal suffering and to reduce the number of animals used. Animals were housed under standard laboratory conditions and monitored daily for health and welfare. At the end of the experimental period, rats were deeply anaesthetised with ketamine before intracardiac blood collection, and euthanasia was completed by exsanguination under deep anaesthesia. Death was confirmed before tissue disposal.

### 2.5. Statistical Analysis

Research data were analysed using the IBM SPSS Statistics version 26.0 (IBM Corp., Armonk, NY, USA) software package. The suitability of the data for a normal distribution was assessed using the Shapiro–Wilk test, whilst homogeneity of variance was evaluated using the Levene test. A repeated measures ANOVA was used to analyse weekly changes in body weight, whilst a one-way ANOVA and Tukey post hoc test were applied for between-group comparisons. Results are presented as mean ± standard deviation, and a *p*-value of <0.05 was considered statistically significant.

## 3. Results

At the start of the experiment, no statistically significant differences were found between the groups in terms of body weight, body mass index or Lee index (Table 2).

At the end of the experiment, a statistically significant difference was observed between the groups’ body weights (*p* < 0.001). The highest final body weight was recorded in the EG (4 h) group (303.25 ± 2.91 g), whilst the lowest final body weight was observed in the exercise group (220.50 ± 3.16 g). Whilst final body weights in the groups exposed to exhaust fumes were found to be significantly higher than those in the control group, this increase was observed to be significantly reduced in the groups subjected to exercise (Table 3). In terms of weight gain, the highest increase during the experiment was observed in the EG (4 h) group (115.62 g), followed by the EG (2 h) group (106.25 g). In contrast, the lowest weight gain was observed in the exercise group (31.50 g). Percentage weight gain values also showed a similar trend, calculated as 61.62% in the EG (4 h) group, 56.47% in the EG (2 h) group, and 16.67% in the exercise group. It was observed that the exercise regimen significantly reduced weight gain associated with exhaust gas (Table 3). At the end of the experiment, significant differences were identified between the groups in terms of BMI, the -Lee index and the visceral adipose index (VAI) values (*p* < 0.001). BMI values were found to be 0.80 ± 0.05 and 0.77 ± 0.04 in the EG (4 h) and EG (2 h) groups, respectively, and were found to be significantly higher than in the other groups. Similarly, Lee index values increased in the groups exposed to exhaust fumes. The VAI value, an indicator of visceral fat, was highest in the EG (4 h) group (1.60 ± 0.07) and lowest in the exercise group (0.80 ± 0.03). The exercise programme significantly reduced BMI, Lee index and VAI values in the groups exposed to exhaust fumes (Table 3).

When evaluated in terms of serum lipid profile, statistically significant differences were identified between the groups (*p* < 0.001) (Table 4). HDL levels decreased significantly in the groups exposed to exhaust fumes, whilst LDL, total cholesterol and triglyceride levels increased significantly. The highest HDL level was observed in the control group (43.51 ± 0.88 mg/dL), whilst the lowest HDL levels were recorded in the EG (4 h) and EG (2 h) groups at 31.21 ± 0.75 mg/dL and 30.79 ± 0.88 mg/dL, respectively. In contrast, LDL levels increased by approximately twofold in the groups exposed to exhaust fumes. Total cholesterol and triglyceride levels were also found to be highest in the EG (4 h) group. The exercise intervention led to an increase in HDL levels whilst causing a marked decrease in LDL, total cholesterol and triglyceride levels (Table 4).

When the results regarding glucose metabolism were examined, it was observed that exposure to exhaust fumes significantly increased serum glucose levels and reduced insulin levels (*p* < 0.001). The highest glucose level was observed in the EG (4 h) group (197.25 ± 5.75 mg/dL), whilst the lowest glucose level was found in the control group (116.75 ± 4.23 mg/dL). Whilst insulin levels were similar in the control and exercise groups, they were significantly reduced in the groups exposed to exhaust fumes. The exercise intervention reduced glucose levels and resulted in a partial improvement in insulin levels (Table 4).

Significant differences were observed between groups in adipokine analyses (*p* < 0.001). Leptin levels increased markedly in response to exhaust gas exposure, with the highest value (5.43 ± 0.43 ng/mL) observed in the EG (4 h) group. In contrast, leptin levels in all groups that underwent exercise were found to be similar to those in the control group. Adiponectin levels, however, increased significantly in the groups exposed to exhaust fumes, with the highest value recorded in the EG (4 h) group (7.18 ± 0.61 mg/L). Although exercise partially reduced adiponectin levels, it was observed that adiponectin levels in the EG (2 h) + E group, in particular, remained at levels similar to those in the groups exposed to exhaust fumes (Table 4).

Statistically significant differences were observed between the groups in terms of inflammation markers (*p* < 0.001) (Table 5). CRP levels were markedly elevated in the groups exposed to exhaust fumes, with the highest value recorded in the EG (4 h) group (21.00 ± 1.45 mg/L). Although the exercise intervention significantly reduced CRP levels, the values did not fully return to those of the control group.

Similarly, IL-1β, IL-6 and TNF-α levels were found to be significantly higher in the groups exposed to exhaust fumes compared to the control group. The highest levels of IL-1β (14.10 ± 0.53 ng/mL), IL-6 (3.43 ± 0.08 ng/L) and TNF-α (225.69 ± 13.85 ng/L) were detected in the EG (4 h) group. In the exercise-treated groups, the levels of these pro-inflammatory cytokines decreased significantly, but remained above control values, particularly in the four-hour exposure group. Levels of IL-37, which possesses anti-inflammatory properties, also showed significant differences between groups (*p* < 0.001). The highest IL-37 level was observed in the EG (4 h) group (634.59 ± 21.49 ng/L), whilst a decrease in IL-37 levels was noted following exercise. In particular, IL-37 levels in the EG (2 h) + E group approached those of the control group. Overall, it was observed that exposure to diesel exhaust from traffic increases systemic inflammation, that the inflammatory response intensifies as the duration of exposure increases, and that regular moderate-intensity exercise significantly suppresses this inflammatory process.

## 4. Discussion

This study demonstrated that traffic-related diesel exhaust exposure induces a comprehensive metabolic disorder in rats, characterised by increased body weight, visceral adiposity, impaired glucose homeostasis, an atherogenic lipid profile, adipokine imbalance, and systemic inflammation. Furthermore, it was determined that regular moderate-intensity exercise significantly reduced these adverse metabolic changes. When the findings are considered together, it is evident that diesel exhaust exposure is not merely an environmental stressor affecting the balance between energy intake and expenditure; rather, it establishes a multifaceted pathophysiological network involving metabolic organs such as adipose tissue, the liver, the pancreas, the hypothalamus and skeletal muscle. The recent characterisation of PM2.5 exposure as an environmental metabolic disruptor playing a role in the development of cardiometabolic diseases further highlights the significance of these findings [12,29,30]. In particular, it is becoming increasingly evident that the metabolic effects associated with air pollution cannot be explained solely by local lung inflammation, but rather involve a combined role of systemic inflammation, oxidative stress, epigenetic changes, mitochondrial dysfunction and neuroendocrine disorders. Mechanistically, these processes may interact to promote obesity through disruption of energy homeostasis. The oxidative potential of particulate matter is particularly relevant in this process, as redox-active constituents, including transition metals and organic compounds, can contribute to ROS generation and depletion of endogenous antioxidant defences. Because different pollutant constituents may contribute through distinct and interacting redox pathways, the overall oxidative burden may reflect their combined effects rather than the action of a single component in isolation [31,32,33]. Pollutant-induced oxidative stress and systemic inflammation can impair the function of metabolically active tissues, including adipose tissue, the liver, skeletal muscle and the hypothalamus. In adipose tissue, these alterations may favour adipocyte dysfunction and lipid accumulation, while mitochondrial impairment may reduce metabolic efficiency and energy utilisation. At the systemic level, inflammatory and neuroendocrine disturbances may further impair glucose and lipid metabolism and disrupt the regulation of energy balance. Collectively, these interconnected alterations can create a metabolic environment that favours visceral fat accumulation, progressive metabolic dysfunction and ultimately obesity [12,29,34].

In our study, the marked weight gain and increase in obesity markers observed in rats exposed to exhaust fumes suggest that PM2.5 exposure disrupts energy homeostasis. Xu et al. (2011) demonstrated that long-term PM2.5 exposure in mice leads to an increase in body weight accompanied by insulin resistance and inflammation of adipose tissue [12]. Similarly, adipocyte hypertrophy, expansion of white adipose tissue and marked impairments in lipid metabolism have been reported in young mice exposed to real-world air pollution [35]. Furthermore, it has been shown that diesel exhaust particles suppress mitochondrial biogenesis in adipose tissue and reduce energy utilisation efficiency [36,37]. When these findings are considered together, it is thought that the weight gain observed in our study reflects not only an increase in fat storage but also a reduction in energy expenditure and functional changes occurring in adipose tissue. The observation of more pronounced metabolic impairment, particularly in the four-hour exposure group, suggests that PM2.5 exposure may exhibit cumulative and dose-dependent effects. Several interconnected biological mechanisms may explain the development of obesity following exposure to air pollutants. PM2.5-induced oxidative stress and systemic inflammation can extend to metabolically active tissues and disrupt both peripheral and central regulation of energy homeostasis. At the peripheral level, oxidative stress and inflammatory signalling promote adipose tissue dysfunction, mitochondrial impairment, adipocyte hypertrophy and white adipose tissue expansion. In parallel, impaired mitochondrial function and suppression of brown adipose tissue thermogenesis can reduce energy expenditure, thereby favouring positive energy balance and lipid accumulation. At the central level, PM2.5-induced hypothalamic inflammation may impair leptin and insulin signalling, alter appetite regulation and further decrease energy expenditure. Moreover, experimental evidence indicates that diesel exhaust particulate matter can modify hypothalamic appetite-related signalling and gut microbiota composition and metabolism, providing an additional gut–brain pathway through which traffic-related pollutants may promote weight gain. Collectively, these mechanisms suggest that pollutant-induced obesity may result from the convergence of oxidative stress, chronic inflammation, impaired adipose tissue thermogenesis and mitochondrial function, altered central energy regulation, and gut–brain metabolic disturbances [38,39].

One of the most striking aspects of weight gain is the marked increase in visceral fat. Today, visceral adipose tissue is recognised as an active endocrine organ at the heart of metabolic syndrome [40]. It has been reported that exposure to PM2.5 increases macrophage infiltration in white adipose tissue, elevates inflammatory gene expression, and leads to adipocyte hypertrophy [12,36,41]. Warren et al. (2024) found that diesel exhaust particles increase inflammatory markers in adipose tissue and impair mitochondrial function [36]. Chen et al. (2023), on the other hand, reported that PM2.5 exposure leads to triglyceride accumulation and suppressed thermogenesis in adipose tissue [42]. Furthermore, it has been found that PM2.5 exposure reduces energy expenditure by decreasing UCP1 expression in brown adipose tissue, thereby facilitating the development of obesity [43,44]. In our study, the fact that CRP, IL-1β, IL-6 and TNF-α levels reached their highest values in the four-hour exhaust exposure group supports the relationship between the increase in visceral adipose tissue and systemic inflammation. Previous experimental studies have also reported that PM2.5 and diesel exhaust particles increase pro-inflammatory cytokine production, activate oxidative stress responses, and induce inflammation in metabolic tissues [45,46,47,48,49,50]. Excessive ROS generation can further amplify inflammatory signalling and mitochondrial dysfunction, thereby creating a self-perpetuating interaction between oxidative stress and metabolic inflammation [33,51]. Consequently, the increase in VAI observed in our study may reflect not only an increase in fat mass but also the development of an inflammatory-active adipose tissue phenotype.

The expansion of visceral adipose tissue, accompanied by macrophage infiltration and increased cytokine production, forms the basis of the process defined as metabolic inflammation [52,53]. In this context, the increases in CRP, IL-1β, IL-6 and TNF-α observed in our study indicate that PM2.5 exposure is not merely an environmental factor that increases fat storage; it also creates a metabolically active inflammatory environment. This inflammatory profile suggests that PM2.5 exposure does not merely cause pulmonary toxicity but also contributes to the development of systemic low-grade chronic inflammation [45,46,47,49].

These changes occurring in adipose tissue are also reflected in the adipokine profile. In our study, the marked increase in leptin levels is consistent with an increase in visceral adipose tissue. However, the rise in leptin is not merely an indicator of fat mass. It is known that increased levels of TNF-α and IL-6 act as a bridge between chronic inflammation and leptin resistance by affecting leptin synthesis and leptin signalling [54]. It has been demonstrated that PM2.5 exposure disrupts leptin signalling and leads to leptin resistance by inducing hypothalamic inflammation [24,55]. Similarly, Liu et al. (2024) reported that diesel exhaust particles activate orexigenic signalling pathways, alter the gut microbiota, and increase the expression of hypothalamic genes that stimulate appetite [56]. When these findings are considered together, it is thought that the increase in leptin observed in our study may be indicative not only of adipocyte expansion but also of a dysfunction in inflammation-mediated central energy regulation.

Findings regarding adiponectin, however, point to a more complex metabolic response. Although many studies on PM2.5 exposure have reported a decrease in adiponectin levels [12,20,29], adiponectin levels were elevated in our study. Whilst this may appear to contradict the literature at first glance, different adiponectin responses have been identified depending on the stage of metabolic stress. In particular, it has been suggested that an increase in adiponectin under conditions of early-stage inflammation and oxidative stress may represent a protective or compensatory mechanism [57]. Similarly, it is noteworthy that levels of IL-37, which possesses anti-inflammatory properties, were also significantly elevated in the exhaust groups in our study. The increase in IL-37 may reflect an endogenous counter-regulatory response to rising levels of IL-1β, IL-6 and TNF-α. However, the persistence of obesity, hyperglycaemia and dyslipidaemia despite the rise in adiponectin and IL-37 levels indicates that these protective mechanisms cannot fully counterbalance the inflammatory and metabolic burden caused by PM2.5 exposure [58,59,60]. In other words, the observed increase in adiponectin and IL-37 levels, despite a marked rise in pro-inflammatory markers, suggests that the organism has developed an adaptive response aimed at rebalancing its disrupted metabolic homeostasis. However, the fact that this response cannot completely prevent metabolic disorders suggests that the inflammatory burden caused by PM2.5 exposure may exceed the capacity of biological defence mechanisms.

It is evident that changes in adipokines are reflected in glucose metabolism. In our study, glucose levels increased whilst insulin levels decreased in rats exposed to exhaust fumes. It has long been known that PM2.5 exposure leads to insulin resistance [61]. Findings from recent years indicate that the metabolic disorders caused by PM2.5 exposure do not manifest solely in adipose tissue; rather, they develop as a systemic pathophysiological process involving the liver, pancreas and other metabolic organs [29]. In particular, it is known that pro-inflammatory cytokines such as IL-1β, IL-6 and TNF-α impair insulin signalling, thereby reducing glucose utilisation and increasing insulin resistance [62]. Lu et al. (2025) demonstrated that subacute PM2.5 exposure increases oxidative stress and inflammation in the liver, thereby inhibiting the PI3K/AKT signalling pathway and causing hepatic insulin resistance [34]. Li et al. (2024) reported that the combination of PM2.5 and a high-fat diet increases fasting glucose and HOMA-IR values [63]. Furthermore, Bosch et al. (2023) demonstrated that particles absorbed via the gut can affect insulin secretion by impairing β-cell function [59]. These findings suggest that the hyperglycaemia and reduced insulin levels observed in our study cannot be explained by a single organ alone; rather, they are the result of a complex metabolic interaction mediated by inflammation between adipose tissue, the liver, the pancreas and the gut. It is known that cytokines such as TNF-α and IL-1β can reduce glucose utilisation by suppressing insulin receptor signalling [64]. Therefore, the hyperglycaemia and reduced insulin levels observed in our study may be related not only to changes in energy metabolism but also to inflammation-induced insulin resistance [34,58].

It is to be expected that disturbances in glucose metabolism will affect lipid metabolism [65]. In our study, the decrease in HDL levels alongside increases in LDL, total cholesterol and triglyceride levels indicates that diesel exhaust exposure creates a distinct atherogenic profile [66]. It has been reported that PM2.5 exposure suppresses fatty acid oxidation in the liver, increases lipogenesis and disrupts lipoprotein metabolism [34,63]. Furthermore, it is known that chronic inflammation affects hepatic lipid metabolism, thereby exacerbating atherogenic dyslipidaemia. Gao et al. (2024) found that exposure to diesel and petrol exhaust disrupts lipid metabolism, and that this effect becomes more pronounced with age [67]. Furthermore, it has been reported that real-world PM2.5 exposure leads to mitochondria-mediated disruptions in lipid metabolism and increases triglyceride accumulation in adipose tissue [35]. Therefore, it is considered that the dyslipidaemia in our study is part of a common metabolic disorder developing alongside inflammation, adipose tissue dysfunction and insulin resistance.

The protective effects of exercise are one of the most significant findings of the study. In the exercise groups, significant improvements were observed not only in body weight, visceral adiposity, glucose levels and lipid parameters, but also in CRP, IL-1β, IL-6 and TNF-α levels. Marmett et al. (2022) noted that PM2.5 exposure partially suppressed some of the positive metabolic adaptations of exercise, but that exercise continued to reduce inflammation and oxidative stress [68]. Qin et al. (2021) reported that aerobic exercise reduces PM2.5-induced inflammation [69]. Fan et al. (2024) found that regular exercise reverses PM-induced metabolic damage via the SIRT1/AMPKα/PGC1-α/NRF1 pathways and alleviates insulin resistance [70]. It is known that exercise supports mitochondrial biogenesis, increases fatty acid oxidation and suppresses the inflammatory response [71,72]. When these mechanisms are considered together, the lower levels of inflammation, lower leptin concentrations, better glucose control and healthier lipid profile observed in the exercise groups in our study appear to be biologically meaningful. However, the fact that metabolic parameters did not return entirely to control levels indicates that the biological burden caused by chronic diesel exhaust exposure is significant and that exercise can only partially reverse these effects.

In conclusion, the findings suggest that diesel exhaust exposure increases systemic inflammation, leading to dysfunction in visceral adipose tissue and adipokine imbalance; this transforms into a comprehensive chain of metabolic dysfunction affecting glucose homeostasis, lipid metabolism and energy regulation. Exercise has demonstrated a protective effect at many stages of this chain; however, it has not been able to completely eliminate the effects of exposure. Therefore, reducing traffic-related air pollution and promoting regular physical activity should be considered complementary strategies in the prevention of obesity and metabolic diseases.

Therefore, environmental interventions aimed at reducing air pollution should be considered as important public health strategies for the prevention of obesity and metabolic diseases, just as much as the promotion of regular physical activity.

## 5. Conclusions

The findings of this study demonstrate that chronic exposure to traffic-related diesel exhaust promotes obesity development and induces a broad spectrum of metabolic disturbances in rats. Diesel exhaust exposure was associated with increased body weight gain, visceral adiposity, dyslipidaemia, impaired glucose homeostasis, adipokine imbalance, and elevated inflammatory markers. The concurrent increase in CRP, IL-1β, IL-6, and TNF-α levels suggests that systemic low-grade inflammation may represent a key biological mechanism linking traffic-related air pollution to metabolic dysfunction and obesity development.

Moderate-intensity exercise substantially attenuated these adverse effects by reducing weight gain, improving metabolic parameters, restoring adipokine balance, and suppressing inflammatory responses. However, exercise did not completely reverse all alterations induced by prolonged diesel exhaust exposure, indicating that the metabolic burden associated with chronic air pollution may persist despite regular physical activity.

Overall, these findings support the growing evidence that traffic-related air pollution should be considered not only a respiratory and cardiovascular risk factor but also a potential contributor to obesity and metabolic disorders. Strategies aimed at reducing exposure to traffic-related air pollution, together with the promotion of regular physical activity, may play complementary roles in mitigating the metabolic health consequences associated with environmental pollution.

### Limitations

Several limitations should be considered when interpreting the findings of this study. First, this investigation was conducted using an experimental rat model; therefore, caution is warranted when extrapolating these findings directly to human populations, where exposure patterns, lifestyle factors, and biological responses may differ substantially. Second, only female Wistar Albino rats were included in the study. Consequently, potential sex-related differences in susceptibility to traffic-related air pollution and exercise responsiveness could not be evaluated. Third, although the exposure protocol was designed to mimic chronic traffic-related air pollution exposure under controlled experimental conditions, the eight-week exposure period may not fully reflect the cumulative effects of long-term environmental exposure experienced throughout the lifespan. Fourth, the study focused primarily on anthropometric, metabolic, adipokine, and inflammatory outcomes and did not investigate the molecular signalling pathways underlying the observed alterations. Therefore, the precise biological mechanisms linking diesel exhaust exposure to obesity development and metabolic dysfunction remain to be clarified. Finally, the controlled laboratory setting does not completely reproduce the complexity of real-world traffic-related air pollution, which consists of varying pollutant mixtures, concentrations, and environmental co-exposures. Future studies incorporating both mechanistic analyses and longer-term exposure models are warranted to further elucidate the pathways through which traffic-related air pollution influences metabolic health.

In addition, gas-phase co-pollutants such as NOx and CO were not quantified during diesel exhaust exposure; therefore, the relative contribution of particulate and gaseous components to the observed effects could not be distinguished.

## Figures and Tables

**Table 1:** Experimental groups and intervention protocols.

Experimental Group	Chamber/Exposure Condition	PM_2.5_ Concentration	Daily Exposure	Exercise Protocol
Control	HEPA-filtered clean air	—	4 h/day	No exercise
Exhaust gas (4 h)	Diesel exhaust	300 μg PM_2.5_/m^3^	4 h/day	No exercise
Exhaust gas (2 h)	Diesel exhaust	300 μg PM_2.5_/m^3^	2 h/day	No exercise
Exercise	HEPA-filtered clean air	—	4 h/day	15 m/min, 30 min/day
Exhaust gas (4 h) + exercise	Diesel exhaust	300 μg PM_2.5_/m^3^	4 h/day	15 m/min, 30 min/day, after exposure
Exhaust gas (2 h) + exercise	Diesel exhaust	300 μg PM_2.5_/m^3^	2 h/day	15 m/min, 30 min/day, after exposure

**Table 2:** Comparison of baseline characteristics of the experimental groups.

Variable	Control	EG (4 h)	EG (2 h)	Exercise	EG (4 h) + E	EG (2 h) + E	*p*
Initial weight (g)	185.63 ± 0.92	187.63 ± 1.92	188.13 ± 3.09	189.00 ± 2.40	189.75 ± 3.45	190.25 ± 2.25	0.073
BMI (initial measurement)	0.49 ± 0.01	0.50 ± 0.02	0.49 ± 0.02	0.49 ± 0.02	0.50 ± 0.02	0.50 ± 0.01	0.083
Lee index (initial measurement)	0.27 ± 0.01	0.28 ± 0.01	0.27 ± 0.01	0.27 ± 0.01	0.27 ± 0.01	0.27 ± 0.01	0.171

Data are presented as mean ± standard deviation. A one-way ANOVA test was used for comparisons between groups. EG: exhaust gas exposure; E: exercise.

**Table 3:** Comparison of anthropometric indicators associated with obesity at the end of the experiment.

**Variable**	**Control**	**EG (4 h)**	**EG (2 h)**	**Exercise**	**EG (4 h) + E**	**EG (2 h) + E**	***p***
Final weight (g)	234.63 ± 1.06 ^b^	303.25 ± 2.91 ^f^	294.38 ± 3.20 ^e^	220.50 ± 3.16 ^a^	262.13 ± 5.22 ^d^	254.75 ± 1.67 ^c^	<0.001
Weight gain (g)	49.00	115.62	106.25	31.50	72.38	64.50	-
Percentage increase in weight (%)	26.40	61.62	56.47	16.67	38.14	33.90	-
BMI (last measurement)	0.59 ± 0.01 ^b^	0.80 ± 0.05 °C	0.77 ± 0.04 ^c^	0.55 ± 0.01 ^a^	0.58 ± 0.02 ^b^	0.60 ± 0.01 ^c^	<0.001
Lee index (final measurement)	0.28 ± 0.01 ^a^	0.32 ± 0.02 ^c^	0.32 ± 0.01 ^c^	0.28 ± 0.02 ^a^	0.29 ± 0.02 ^b^	0.29 ± 0.01 ^b^	<0.001
VAI	0.91 ± 0.07 ^e^	1.60 ± 0.07 ^a^	1.41 ± 0.06	0.80 ± 0.03 ^f^	1.32 ± 0.05 ^c^	1.23 ± 0.03 ^c^	<0.001

Data are presented as mean ± standard deviation. Group means with different superscript letters in the same row show statistically significant differences according to the Tukey post hoc test (*p* < 0.05). BMI: body mass index; VAI: visceral adipose index; EG: exhaust gas exposure; E: exercise.

**Table 4:** Comparison of serum metabolic parameters according to experimental groups.

	Control	EG (4 h)	EG (2 h)	Exercise	EG (4 h) + E	EG (2 h) + E	*p*
Parameters	X ± SD	X ± SD	X ± SD	X ± SD	X ± SD	X ± SD	
HDL (mg/dL)	43.51 ± 0.88 ^a^	31.21 ± 0.75 ^d^	30.79 ± 0.88 ^d^	40.50 ± 4.77 ^b^	35.55 ± 0.95 ^c^	35.74 ± 1.86 ^c^	<0.001
LDL (mg/dL)	6.95 ± 0.26 ^d^	13.30 ± 0.43 ^a^	13.48 ± 0.36 ^a^	6.77 ± 0.15 ^d^	8.21 ± 0.30 ^b^	7.47 ± 0.32 ^c^	<0.001
Cholesterol (mg/dL)	49.75 ± 3.11 ^dc^	76.25 ± 3.24 ^a^	66.38 ± 2.56 ^b^	46.83 ± 3.14 ^d^	51.57 ± 5.83 ^c^	52.00 ± 1.60 ^c^	<0.001
Triglycerides (mg/dL)	128.25 ± 3.96 ^e^	192.88 ± 5.11 ^a^	181.25 ± 6.78 ^b^	72.67 ± 3.65 ^f^	158.75 ± 5.01 ^c^	151.38 ± 3.0 ^d^	<0.001
Glucose (mg/dL)	116.75 ± 4.23 ^e^	197.25 ± 5.75 ^a^	186.50 ± 6.91 ^b^	121.13 ± 5.06 ^e^	152.50 ± 4.75 ^c^	142.00 ± 4.41 ^d^	<0.001
Insulin (μg/L)	4.09 ± 0.01 ^a^	2.07 ± 0.06 ^c^	2.03 ± 0.04 ^c^	4.06 ± 0.04 ^a^	3.07 ± 0.03 ^b^	3.05 ± 0.03 ^b^	<0.001
Adiponectin (mg/L)	4.78 ± 0.31 ^d^	7.18 ± 0.61 ^a^	6.25 ± 0.45 ^b^	4.84 ± 0.31 ^d^	5.45 ± 0.48 ^c^	6.00 ± 0.40 ^b^	<0.001
Leptin (ng/mL)	2.53 ± 0.16 ^c^	5.43 ± 0.43 ^a^	3.80 ± 0.32 ^b^	2.79 ± 0.52 ^c^	2.56 ± 0.21 ^c^	2.58 ± 0.20 ^c^	<0.001

There is a statistically significant difference between the group means bearing different superscript letters in the same row according to the Tukey post hoc test (*p* < 0.05).

**Table 5:** Comparison of inflammation markers across experimental groups.

	Control Group	EG (4 h) Group	EG (2 h) Group	E Group	EG (4 h) + E Group	EG (2 h) + E Group	*p*
	X ± SD	X ± SD	X ± SD	X ± SD	X ± SD	X ± SD	
CRP (mg/L)	5.12 ± 0.62 ^e^	21.00 ± 1.45 ^a^	15.73 ± 0.65 ^b^	5.77 ± 0.65 ^e^	14.67 ± 0.56 ^c^	12.09 ± 1.29 ^d^	<0.001
IL-37 (ng/L)	452.19 ± 38.00 ^d^	634.59 ± 21.49 ^a^	544.10 ± 22.46 ^b^	459.65 ± 18.60 ^d^	498.59 ± 24.95 ^c^	465.80 ± 25.90 ^d^	<0.001
IL-1β (ng/mL)	8.37 ± 0.62 ^d^	14.10 ± 0.53 ^a^	12.28 ± 0.50 ^b^	8.77 ± 0.58 ^d^	12.16 ± 0.41 ^b^	10.56 ± 0.93 ^c^	<0.001
IL-6 (ng/L)	1.92 ± 0.10 ^c^	3.43 ± 0.08 ^a^	3.08 ± 0.16 ^a^	2.67 ± 0.13 ^b^	2.61 ± 0.38 ^b^	2.11 ± 0.79 ^c^	<0.001
TNF-α (ng/L)	132.81 ± 3.34 ^d^	225.69 ± 13.85 ^a^	195.28 ± 4.54 ^b^	166.80 ± 11.04 ^c^	193.89 ± 5.60 ^b^	164.66 ± 4.22 ^c^	<0.001

Data are presented as mean ± standard deviation. There is a statistically significant difference (*p* < 0.05) between group means denoted by different superscript letters in the same row, as determined by the Tukey post hoc test. CRP: C-reactive protein; IL-37: interleukin-37; IL-1β: interleukin-1 beta; IL-6: interleukin-6; TNF-α: tumour necrosis factor alpha; EG: exhaust gas exposure; E: exercise.

## Data Availability

The datasets generated and/or analysed during the current study are available from the corresponding author on reasonable request.
